# Pulmonary function in children with idiopathic scoliosis

**DOI:** 10.1186/1748-7161-7-7

**Published:** 2012-03-23

**Authors:** Theofanis Tsiligiannis, Theodoros Grivas

**Affiliations:** 1 Pediatric Pulmonology, Mitera Pediatric Hospita, Kifisias & Erythrou Stavrou Street 6, Amarousion, 15123 Athens, Greece; 2 Office address: Theofanis Tsiligiannis MD, Aegialias 30 Amarousion, 15125 Athens, Greece; 3 Department of Trauma and Orthopaedics, "Tzanio" General Hospital of Piraeus, Tzani & Afendouli str, 18536 Piraeus, Greece

## Abstract

Idiopathic scoliosis, a common disorder of lateral displacement and rotation of vertebral bodies during periods of rapid somatic growth, has many effects on respiratory function. Scoliosis results in a restrictive lung disease with a multifactorial decrease in lung volumes, displaces the intrathoracic organs, impedes on the movement of ribs and affects the mechanics of the respiratory muscles. Scoliosis decreases the chest wall as well as the lung compliance and results in increased work of breathing at rest, during exercise and sleep. Pulmonary hypertension and respiratory failure may develop in severe disease. In this review the epidemiological and anatomical aspects of idiopathic scoliosis are noted, the pathophysiology and effects of idiopathic scoliosis on respiratory function are described, the pulmonary function testing including lung volumes, respiratory flow rates and airway resistance, chest wall movements, regional ventilation and perfusion, blood gases, response to exercise and sleep studies are presented. Preoperative pulmonary function testing required, as well as the effects of various surgical approaches on respiratory function are also discussed.

## Introduction

Idiopathic scoliosis has direct effects on many aspects of the respiratory function and, although there have been reviews on the subject [1,2], the purpose of this review is a detailed discussion on how scoliosis is affecting different aspects of breathing and especially how it is reflected in the results of the various tests of respiratory function.

## Epidemiological and anatomical aspects

Scoliosis is the most common 3-dimensional deformation abnormality of the spine with direct effects on the thoracic cage, with a reported prevalence in the general population varying significantly from 0.3% to 15.3% [3-6]. It is the result of a pathologic process which leads to lateral curvature of the spine. It can be congenital, due to vertebral or rib malformation, secondary to a variety of systemic or neuromuscular disorders, or idiopathic. Idiopathic scoliosis accounts for approximately 85% of cases and it is defined as a structural scoliosis for which no specific cause can be established [7]. Based on population studies it is considered a single-gene disease with variable penetrance and heterogeneity [8,9]. Idiopathic scoliosis is further categorized as infantile, juvenile and adolescent, according to the age at which the spinal deformity is first noticed. Its diagnosis can be made after exclusion of a primary etiology such as vertebral anomaly, neuromuscular disorder, Marfan syndrome or other disorder. Another classification is based on the quantification of the severity of the scoliosis by the use of radiographic measurements of the angle of the curvature in the spine (Cobb angle), as well as the level of the apex of the spinal curvature (cervical, high thoracic, thoracolumbar, lumbar), and the number of curves (single or double). These characteristics are used for comparison, prognosis and development of treatment guidelines.

The effects of scoliosis in the anatomy of the chest are quite complex. It was reported that the plot of segmental thoracic ratios by age groups, a method of assessment of the morphology of rib cage in anteroposterior radiographs, is one way of expressing the proportional changes of thoracic viscera, mainly the heart and lungs and principally the lungs during growth. The relative narrowing of the girls' lower thorax between childhood and puberty is consistent with the proportionate change in the girl's lung in the later stages of growth reported by Simon et al [10,11]. The narrowing of the girls' lower thorax between childhood and puberty is also documented assessing the segmental rib-vertebra angles (RVAs) in Segmental patterns of chest radiographs in children [12,13].

The upper chest in Infantile Idiopathic Scoliosis (IIS) is funnel - shaped. It was reported that the narrow rib - cage at T1-4 in children with IIS compared with the controls is very highly significant [14]. The funnel - shaped upper thoracic cage of IIS is like that of each of: a) a normal human foetus, b) asphyxiating thoracic dysplasia (Jeune's disease) [15] and c) a normal adult rabbit. The rib cage in Adolescent Idiopathic Scoliosis (AIS) patients was also documented to be narrower than in the non scoliotic counterparts [16]. Consequently if the chest cannot develop normally during growth, there is insufficient space available for pulmonary alveolar growth, with resultant extrinsic restrictive lung disease [17-19].

Scoliosis can affect pulmonary function in many ways. At an early stage it is usually painless and asymptomatic. Most investigators who have studied the impairment of pulmonary function in scoliosis generally agree that (1) a Cobb angle greater than 90 degrees greatly predisposes to cardiorespiratory failure, (2) lung function abnormalities are detectable when a Cobb angle is greater than 50 to 60 degrees, (3) lung function abnormalities are mainly of the restrictive type and (4) the duration of scoliosis correlates with the patients degree of disability [7]. Its natural history is associated with curve progression, cardiopulmonary impairment, back pain, cosmetic deformity and neurologic compromise. A great variety on the degree of these manifestations exists, depending on age of onset, genetic background and curve pattern [7].

## Pathophysiology and effects of idiopathic scoliosis on respiratory function

In the absence of other underlying disorders, mild to moderate scoliosis (Cobb angle less than 70°) actually produces very few respiratory signs and symptoms. Although scoliosis has generally been associated with the development of restrictive lung disease, resulting in decreased lung volume as manifested by a decrease in total lung capacity (TLC) on pulmonary function testing, it can affect respiratory function in many ways.

The decrease in lung volume is multifactorial and may reflect different pathophysiology depending on the age of the patient at the onset of scoliosis and the chronicity of the problem. It is mainly due to restriction which is related to the severity of scoliosis (Cobb angle), the location of the curve, and the loss of normal thoracic kyphosis [20]. Apart from the degrees of the curve, the level of the curve and the amount of spinal rotation are also important in determining the amount of respiratory compromise. The more cephalad the curve, the more severely the lung on the convex side is compressed. Spinal rotation shifts the ribs laterally, so that the midpoint of the sternum is lateral to the midpoint of the spine and this, further compresses, or distorts the lungs, by flattening them in the lateral plane [21].

True lung hypoplasia, due to thoracic deformity during the period of very rapid lung growth and development, may be a factor in infantile and, possibly, juvenile scoliosis [22-24]. Post mortem quantitative studies suggest that alveolar multiplication is decreased in infantile scoliosis, whereas the alveoli may not enlarge normally in juvenile or adolescent scoliosis. The number of pulmonary vessels in scoliotic children is also reduced in proportion to lung maldevelopment [7].

Impaired chest wall mechanics that prevent normal inflation of the lungs results in decreased TLC in the adolescent form, when the development and growth of the lungs has been completed before its onset. Defective mechanical coupling of inspiratory muscles to the chest wall leading to a decrease in respiratory muscle mechanics, has been shown to contribute to the restrictive properties [25]. Long standing hypo-inflation and atelectasis probably leads to irreversible atrophy of the lung and further reduction of lung volume.

Airway obstruction may occur but is uncommon. Rotation of the chest can produce displacement/rotation of the intrathoracic and/or mainstem bronchi, or compression of a mainstem bronchus against vertebra and mediastinal structures, produce mechanical airway obstruction, reduce expiratory flows and increase airway resistance [26,27]. Lower airway obstruction may develop as the disease worsens and is often reversible with bronchodilators. This indicates presence of airway hyper-responsiveness attributed to chronic airway inflammation secondary to poor clearance of secretions [28]. Respiratory muscle "weakness" occurs because of abnormal configuration of the diaphragm rather than a primary myopathy per se. The torsion on the diaphragm may increase the radius of the curvature of the diaphragm, thereby decreasing the force-generating capacity and making it less efficient [21]. The intercostal muscles are also affected by the distortion of the rib cage. Decrease in the anteroposterior diameter of the thorax and actual displacement of the heart may impede on its function by not allowing the increase in stroke volume necessary during conditions of increased metabolic demand.

Breathing pattern is significantly altered in severe scoliosis at rest, on exertion and during sleep. The respiratory rate tends to be higher and the tidal volume lower than normal. Despite though its absolute value, tidal volume is actually increased relative to the vital capacity. To accomplish this, patients need an inspiratory effort that is often more than twice normal, and this is achieved with a much higher than normal trans-diaphragmatic pressure, requiring increased contribution from the abdominal expiratory muscles [29]. Those abdominal muscles assist inhalation by increasing the intra-abdominal pressure during exhalation. These mechanisms increase significantly the work of breathing, and, when used for regular breathing (instead of relatively short periods of time during exercise) increase significantly the risk of respiratory muscle fatigue and respiratory failure.

Disordered breathing during sleep has been described in children with early onset scoliosis in the absence of neuromuscular weakness. Alveolar hypoventilation first occurs at night and patients with scoliosis have been also found to have central hypopnea or even true apnea associated with decreased oxygen saturations especially during rapid eye movement (REM) sleep [29].

## Pulmonary function testing in idiopathic scoliosis

### Lung volumes

Restrictive lung disease manifested by a reduction in the total lung capacity (TLC) is characteristic of severe scoliosis. However, the measurement of the TLC requires equipment (body plethysmography, helium dilution or nitrogen washout) that are not readily available in every clinical setting. In such cases simple spirometry may provide a good estimate of the restrictive lung defect because the decrease in the FVC is proportional to the decrease in TLC unless the patient has a mixed restrictive and obstructive defect. At the very least, complete lung volume measurements should be performed for the pre-operative and post-operative evaluation. Spirometry is much easier to perform in outpatient care settings and is more useful in monitoring the changes in lung function over time, than as the sole means of diagnosing restrictive respiratory disease [30,31]. In patients with moderate to severe scoliosis a negative linear correlation has been established between the magnitude of the curve and FVC. A reduction of lung volumes has also been reported in some adolescents with mild scoliosis (e.g. Cobb angle less than 35 degrees), without a clear correlation between the magnitude of the curve and lung volumes. The FVC decreases in proportion to TLC unless there is air-trapping in which case the FVC decreases disproportionately. Residual volume (RV) remains generally within the predicted values. Due to the relative decrease in TLC, RV/TLC ratio is increased. Similarly, the functional residual capacity (FRC) is also normal or slightly diminished and the FRC/TLC ratio is increased. If scoliosis progresses to a severe degree, RV declines slightly. The absolute values of anatomic and alveolar dead space are believed to remain normal. If there is atelectasis and/or hypoinflation of the lung the alveolar dead space will be decreased as well. However, the ratio of dead space to tidal volume (V_D_/V_T_) is increased. This plays a major role in the development of alveolar hypoventilation.

Maximum inspiratory pressure (MIP) has been reported to be decreased. A crude but significant correlation between the decrease in MIP and the fall in FVC has been reported [3,32]. The decrease in MIP is of major importance in the pre-operative evaluation of the patient because a MIP less than 30 cmH_2_O increases significantly the possibility of postoperative respiratory failure due to inability to get extubated. Maximum expiratory pressure (MEP) is normal or may be low, probably due to the chest wall deformity that prevents the muscles from contracting effectively and thus they can not generate the maximal pressure.

In general, the effects on lung volumes in idiopathic scoliosis are the result of reduction of chest wall compliance, impaired lung growth and impaired respiratory muscle strength which work at a mechanical disadvantage.

### Respiratory flow rates and airway resistance

Expiratory flow rates are decreased proportionally to the restricted lung volume, while FEV1/FVC ratio is normal [7]. Air trapping in the majority of patients was found by measurements of FRC_pleth_/FRC_He _ratios [28]. The lower airway obstruction is occasionally reversible with bronchodilators, indicating presence of airway hyper-responsiveness. This may be the result of chronic airway inflammation secondary to the poor clearance of secretions. The decrease in FRC_pleth _after use of bronchodilators suggests that these patients may have increased bronchomotor tone, perhaps to compensate for the restricted chest wall and to resist the compressive forces from the scoliosis [28]. Airway hyper-responsiveness associated with atopy has to be also considered and must be independently assessed in these patients [28].

Airway resistance may be normal or increased due to airway distortion associated with chest deformity.

### Elastic properties

The compliance of the respiratory system is decreased. In particular, the decreased chest wall compliance plays an important role in the impairment of lung volumes and correlates closely with the severity of scoliosis and with the decrease in FVC [33]. An associated mild reduction of lung compliance has been attributed to compression of the lungs, reorientation of the surface tension forces and/or alteration of elastic properties of the lungs.

### Chest wall movements

Radiological studies have shown that the movements of the diaphragm are greater on the side of the convex spinal curve. Rib cage excursion has been reported to vary considerably from almost no expansion to normal thoracic movement. Gated images of diaphragm and chest wall positions with dynamic magnetic resonance imaging have been used to describe relative excursion of different components of the thorax both at tidal breathing and at TLC [2].

### Regional ventilation and perfusion

Asymmetric ventilation and perfusion between the right and left lungs occurs in more than half of the children with severe congenital and infantile thoracic scoliosis [34]. However, the severity of lung function asymmetry does not relate to Cobb angle measurements. Asymmetry in lung function is influenced by deformity of the chest wall in multiple dimensions, and cannot be ascertained by chest radiographs alone. The lung on the convex side receives a greater volume of alveolar ventilation than the lung on the (smaller) concave side [35]. Apart from the severity of the spinal curve, the age of the patient is an important factor of alveolar gas misdistribution with normal values of ventilation having been found in young patients with rather severe scoliosis. Air misdistribution is more often found in older patients [36].

### Blood gases

Mild hypoxemia with normocapnia has been commonly encountered in patients with idiopathic scoliosis presumptively due to ventilation-perfusion mismatch. In severe scoliosis hypoxemia may be due to diffusion limitation and/or alveolar hypoventilation, and may be accompanied by CO_2 _retention. In one study of 138 children with early onset scoliosis, the serum hemoglobin level was more than 2 standard deviations above normal in 23% of those studied and improved after surgical intervention. It was speculated that this was due to sleep-related hypoventilation and improved after surgical correction [37]. The elevated V_D_/V_T _ratio also contributes to alveolar hypoventilation.

### Response to exercise

Resting ventilation is increased in scoliotic patients, who maintain a normal PaCO2 despite an increased V_D_/V_T _ratio and an abnormal V/Q ratio. During exercise testing, the ventilatory response is lower in scoliotic patients than in normal subjects. Exercise capacity is usually decreased, even in patients with mild scoliosis and dyspnea on exertion may be one of the first clinical manifestations of scoliosis, or the first manifestation of respiratory impairment in the already diagnosed ones. In one study, the 6 minute walk test (6MWT) in 86 patients with adolescent idiopathic scoliosis and a spine curve ranging from 45° to 138°, revealed a significant increase in RR and Borg Scale scores, as well as a decrease in oximetry (SpO_2_) and in the distances walked, when compared with adolescents without spinal deformities [38]. In moderate and more severe scoliotic patients (Cobb angle more than 40 degrees) work capacity is reduced, heart rate is higher per work load and ventilatory reserve is reduced in some but not all patients [32,39]. The strategy of breathing adopted by scoliotic patients during exercise combines a reduced (rather than increased) V_T _with an increased breathing frequency in order to minimize the increase in work of breathing. Reduced respiratory system compliance, increased work of breathing, perhaps a blunted respiratory drive and decreased muscle strength, account for their lower respiratory performances. It is possible that displacement and/or compression of the heart due to thoracic deformity may not allow the increase in stroke volume that is necessary during exercise. In addition, exercise is likely to increase the already elevated pulmonary artery pressure and in patients with a Cobb angle more than 100° degrees, pulmonary hypertension may contribute to decreased exercise capacity. In a recent study of 60 patients with adolescent idiopathic scoliosis and a Cobb angle more than 40° lung and muscle function, together with exercise capacity were assessed [32]. It was found that reduced muscle strength, involving both the respiratory muscles and also the quadriceps muscles correlated with reduced work capacity. Exercise was terminated due to leg discomfort rather than dyspnea. The results showed generalized muscle dysfunction which contributed to the reduction in their exercise capacity, even in the absence of severe ventilatory impairment. The limitations in exercise are ascribed to cardiopulmonary limitations, muscle weakness, and also physical and cardiovascular deconditioning [32,39,40].

### Sleep studies

Disordered breathing during sleep with central hypopnea and/or true apnea associated with desaturations, especially during REM sleep, has been found in scoliotic patients. In one small study, the Apnea-Hypopnea Index (AHI) was abnormal in 14 of 15 patients but could not be explained by apneic events. Instead, hypopneic episodes associated with oxy-hemoglobin desaturation and arousals were common and occurred more frequently in REM sleep [41]. The authors postulated that low lung volumes typical of thoracic cage deformities predisposed children to oxy-hemoglobin desaturation when hypopnea occurs. Currently there is no consensus as to when polysomnograms (PSGs) should be ordered in this group of patients.

## Preoperative and postoperative pulmonary function in idiopathic scoliosis

Pulmonary complications are the principal cause of morbidity and mortality in the immediate period following surgery for scoliosis. Although there is no direct correlation between the preoperative pulmonary function of a patient and the incidence and severity of postoperative complications, preoperative assessment of pulmonary function including TLC and an overnight oximetry should be performed as a guide to prevent postoperative complications. From a practical standpoint the most useful parameters are the vital capacity and the respiratory muscle strength. An FVC less than 40% of the predicted normal and maximal inspiratory and expiratory pressures of less than 30 cm H_2_O significantly increase the risk that the patient may not be able to be extubated [1,42].

Depending on the surgical approach, studies have demonstrated improvement, decline, or no effect on pulmonary function [43-45]. Surgical intervention corrects the spinal curvature, but its effect on lung volume and arterial oxygenation only becomes apparent later after surgery and improvement may not be measurable for 2 years or more [46,47]. Spinal fusion in young children may result in a short trunk and stunted growth of the thorax and lungs [22,48]. Controversy still exists over whether Harrington instrumentation improves lung function. A meta-analysis of 173 patients indicated a significant improvement in lung function of 2% to 11% [49].

An expansion thoracoplasty on the concave side of the scoliosis by means of a vertical, expandable prosthetic titanium rib (VEPTR) was originally designed to treat thoracic insufficiency secondary to congenital anomalies with fused ribs, hypoplastic chest wall deformities, and early-onset scoliosis. Several reports suggest the device may increase thorax and lung volume, as well as hemoglobin levels [21,37,50,51]. In one study with data from 7 different centers, although there was a clinically and radiographically apparent expansion of the thorax after VEPTR insertion, no similar improvement in lung volume was found, and instead a decrease in forced vital capacity and increase in residual volume was documented [52]. In a recent study, serial VPTR expansion thoracoplasty resulted in significant increases in lung volume in most patients as evidenced by increases in FVC over time. The effect appeared to be far better when the initial surgical intervention was performed before the age of 6 years. A decreasing respiratory system compliance was noticed over time which is a major concern for the long term effect of this intervention [48].

In summary, idiopathic scoliosis is a common debilitating deformity of the thoracic cage with potentially severe and irreversible effects on lung function. Because the pulmonary manifestations may not become clinically evident until significant or irreversible changes in lung function have already occurred, early recognition of the problem and regular evaluation with pulmonary function testing are advisable.

## Competing interests

The authors declare that they have no competing interests.

## Authors' contributions

TT contributed in drafting the text and finding and obtaining references. TBG helped in drafting the text and finding and obtaining references. The two authors contributed their professional skills to the ensuing discussions as the text progressed. All authors have read and approved the final manuscript.
